# Supplementary material for: Statins Do Not Directly Inhibit the Activity of Major Epigenetic Modifying Enzymes
Source: Cancers (Basel). 2019 Apr 10;11(4):516. doi: 10.3390/cancers11040516 (PMC6521159; doi:10.3390/cancers11040516)
Supplement: Supplementary file 1 [file cancers-11-00516-s001.pdf]

Supplementary material: Statins do Not Directly Inhibit the Activity of Major Epigenetic Modifying Enzymes

Stephanie Bridgeman, Wendy Northrop, Gaewyn Ellison, Thiruvarutchelvan Sabapathy, Phillip Melton, Philip Newsholme and Cyril Mamotte

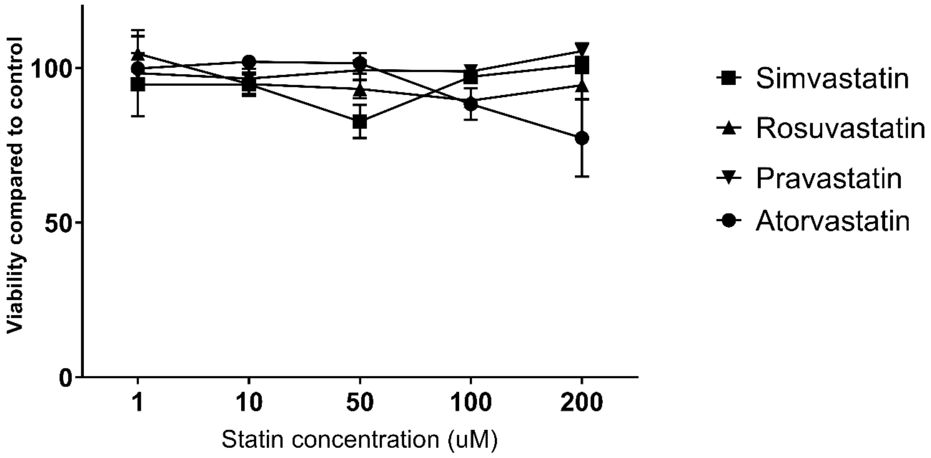

Figure S1. The effect of 24 h statin treatment on the viability of MDA-MB-231 cells.

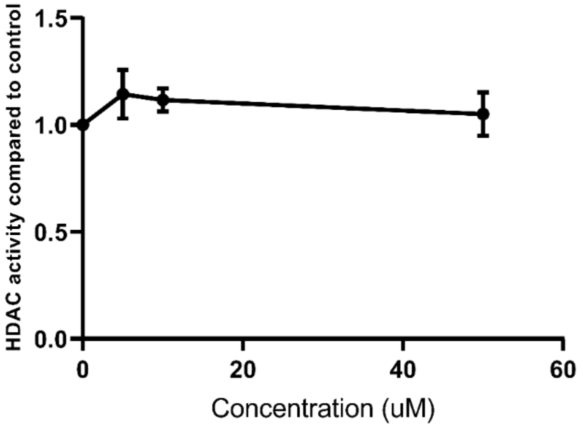

Figure S2. HDAC activity of HepG2 cells following 24 h atorvastatin treatment.

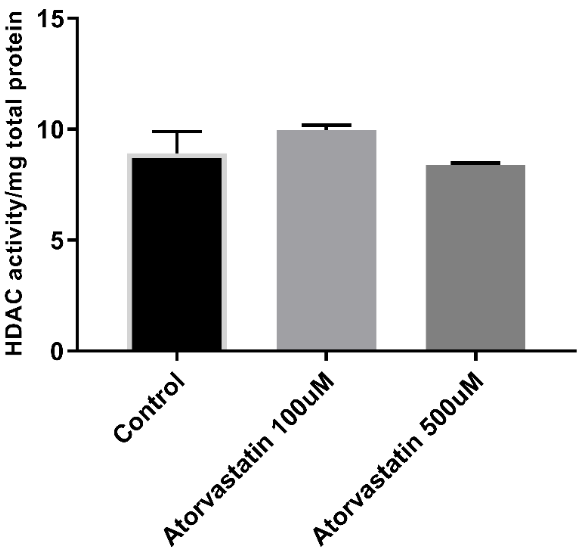

Figure S3. HDAC activity of MDA-MB-231 nuclear extracts.

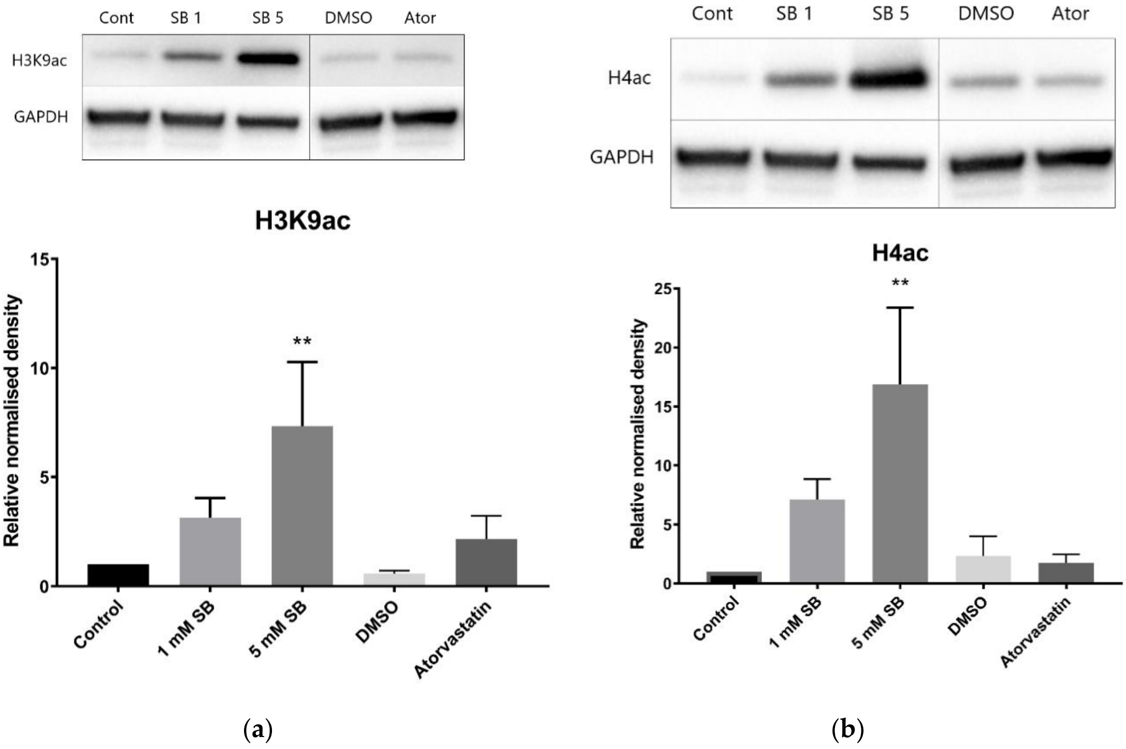

Figure S4. Immunoblotting of BRIN-BD11 cell extracts. (**a**) Relative level of acetylated histone H3K9 in BRIN-BD11 cells; (**b**) relative level of acetylated histone H4 in BRIN-BD11 cells. Cells were treated for 24 h with 1 mM or 5 mM sodium butyrate (SB), 10 μM atorvastatin or equivalent DMSO vehicle control. The graphs illustrate combined density readings normalised to GAPDH from 3 independent experiments. ** $p < 0.01$.

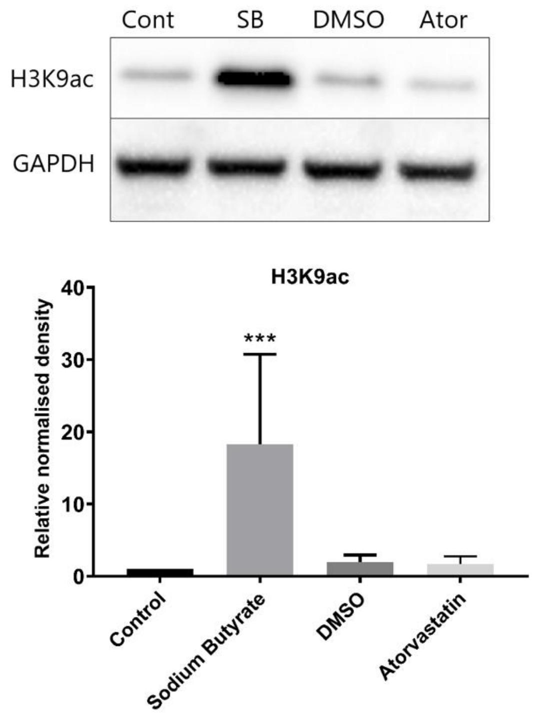

Figure S5. Immunoblotting of extracts of HepG2 cells treated in high lipoprotein serum. Cells were treated for 24 h with 5 mM sodium butyrate (SB), 10 μM atorvastatin or equivalent DMSO vehicle control. The graphs illustrate combined density readings normalised to GAPDH from 3 independent experiments. *** $p < 0.001$.

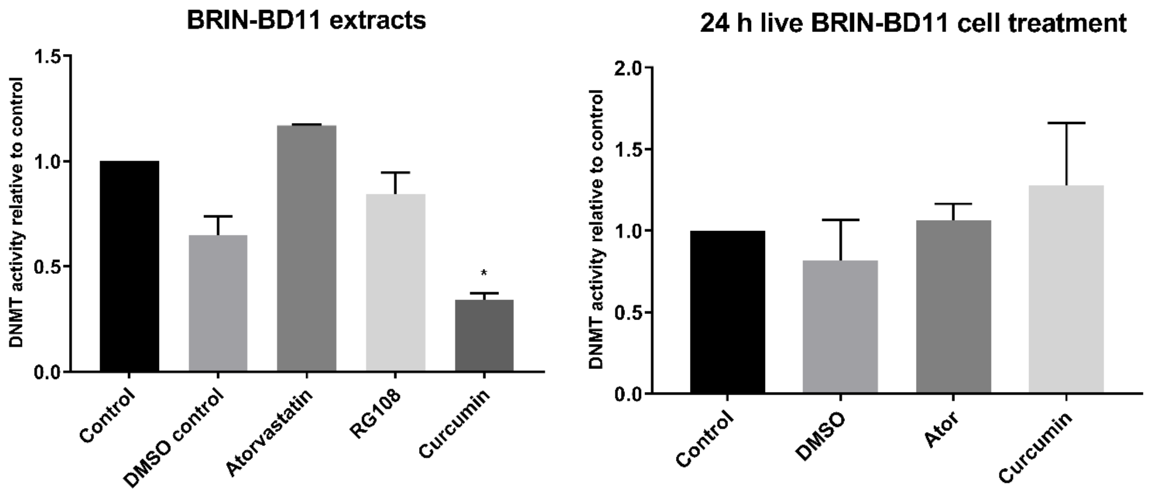

(**a**) (**b**)

Figure S6. DNMT activity of BRIN-BD11 cell extracts. (**a**) DNMT activity in whole cell extracts treated directly with 200 μM atorvastatin, RG-108, curcumin or DMSO vehicle control; (**b**) DNMT activity in whole cell extracts from cells treated for 24 h with 10 μM atorvastatin, curcumin or DMSO control. * $p < 0.05$.

© 2019 by the authors. Licensee MDPI, Basel, Switzerland. This article is an open access article distributed under the terms and conditions of the Creative Commons Attribution (CC BY) license (http://creativecommons.org/licenses/by/4.0/).
